# Endocytosis and Trafficking of Heparan Sulfate Proteoglycans in Triple-Negative Breast Cancer Cells Unraveled with a Polycationic Peptide

**DOI:** 10.3390/ijms21218282

**Published:** 2020-11-05

**Authors:** Elisabetta Mandarini, Eva Tollapi, Marta Zanchi, Lorenzo Depau, Alessandro Pini, Jlenia Brunetti, Luisa Bracci, Chiara Falciani

**Affiliations:** Department of Molecular Biotechnology, University of Siena, Via Aldo Moro 2, 53100 Siena, Italy; elisabetta.mandarini@gmail.com (E.M.); eva.tollapi@student.unisi.it (E.T.); marta.zanchi1@student.unisi.it (M.Z.); lorenzo.depau@unisi.it (L.D.); alessandro.pini@unisi.it (A.P.); jlenia.brunetti@unisi.it (J.B.); luisa.bracci@unisi.it (L.B.)

**Keywords:** heparan-sulfate proteoglycans, endocytosis, breast cancer, vesicular traffic, tumor-targeting peptide

## Abstract

The process of heparan sulfate proteoglycan (HSPG) internalization has been described as following different pathways. The tumor-specific branched NT4 peptide has been demonstrated to bind HSPGs on the plasma membrane and to be internalized in tumor cell lines. The polycationic peptide has been also shown to impair migration of different cancer cell lines in 2D and 3D models. Our hypothesis was that HSPG endocytosis could affect two important phenomena of cancer development: cell migration and nourishment. Using NT4 as an experimental tool mimicking heparin-binding ligands, we studied endocytosis and trafficking of HSPGs in a triple-negative human breast cancer cell line, MDA-MB-231. The peptide entered cells employing caveolin- or clathrin-dependent endocytosis and macropinocytosis, in line with what is already known about HSPGs. NT4 then localized in early and late endosomes in a time-dependent manner. The peptide had a negative effect on CDC42-activation triggered by EGF. The effect can be explained if we consider NT4 a competitive inhibitor of EGF on HS that impairs the co-receptor activity of the proteoglycan, reducing EGFR activation. Reduction of the invasive migratory phenotype of MDA-MB-231 induced by NT4 can be ascribed to this effect. RhoA activation was damped by EGF in MDA-MB-231. Indeed, EGF reduced RhoA-GTP and NT4 did not interfere with this receptor-mediated signaling. On the other hand, the peptide alone determined a small but solid reduction in active RhoA in breast cancer cells. This result supports the observation of few other studies, showing direct activation of the GTPase through HSPG, not mediated by EGF/EGFR.

## 1. Introduction

Heparan sulfate proteoglycans (HSPGs) are a group of glycosylated proteins, classified on the basis of their cell localization or according to the nature of the glycosaminoglycan group attached to the core protein. Glycosaminoglycans (GAGs) are attached to serine residues and are linear acidic polymers designated as heparan sulfate (HS), chondroitin sulfate (CS) and dermatan sulfate (DS) proteoglycans [1].

Membrane HSPGs are syndecans and glypicans and act as receptors or co-receptors for a variety of ligands, particularly growth factors, and are therefore involved in various cell signaling pathways. HSPGs have long HS chains composed of 40–300 sugar residues, many of which are acidic sugars modified by sulfate groups and are, therefore, highly negatively charged [1].

The process of HSPG internalization has been the focus of research in many different fields. So far, various endocytic pathways have been assigned to HSPGs. HSPGs have been described as endocytic receptors for viruses, such as HIV [2], that bind HSPG and are internalized via clathrin-, caveolin- and also macropinocytosis-mediated processes [3], and for other pathogens, such as *Trichomonas vaginalis*, that are internalized in a caveolin-dependent process [4]. Syndecan-1 (SDC1) is a major hepatic receptor for remnant lipoproteins that, upon binding to the HSPG and clustering, are internalized in a flotillin-dependent manner [5]. SDC1 was also recently identified as a regulator of macropinocytosis, an endocytic process that fuels tumor cells [6]. Syndecan-4 (SDC4) and FGF2 are internalized together after binding and receptor activation, employing macropinocytosis [7]. The HSPG endocytic process is currently thought to depend more on the extracellular ligand than on the type of HSPG [8], which is the explanation of why HSPG endocytosis is not confined to a single pathway.

HSPG endosomal trafficking has been much less studied. Around 60 different Rab GTPases have been identified in mammals to date and each family member is typical of a specific endosome [9]. Rab proteins are, therefore, often used as markers of different endosomes. These small proteins of around 200 amino acids bind to a variety of effector proteins involved in vesicle formation, transport and activation of signaling pathways. Rab5a is one of the most studied members of the family; it regulates internalization of receptor tyrosine kinases (RTKs), G protein-coupled receptors (GPCRs) and antigen recognition receptors [10]. Most endocytic pathways have been observed to involve Rab5 and the associated protein, early endosomal antigen-1 (EEA1), although there are different endocytic mechanisms and further sorting to various destinations [11]. During maturation from early to late endosomes, Rab5 is switched by Rab7 on the surface of vesicles [10]. For particles that are recycled or secreted, Rab11 directs and controls the process [12].

The peptide NT4 proved to bind HSPG [13] and to be highly specific for cancer cells, including pancreas and colon adenocarcinoma and bladder and breast cancer [14]. NT4 is a tetrabranched peptide obtained by synthesizing four copies of the 13-amino-acid sequence on a branched core of lysines. Its branched structure imparts stability against proteolytic enzymes [15] and the ability to establish multivalent binding [16,17]. The peptide has already been tested in animal models of cancer and has shown good specificity for tumor versus healthy cells [18]. It also countered the invasive phenotype of breast cancer cells in migration and invasion experiments and reduced angiogenesis of endothelial cells [19].

NT4 is internalized in cancer cells after binding with HSPGs [13]. Here, we used NT4 to explore internalization and trafficking of HSPGs and to unravel the HSPG-activated signaling pathways that may be involved in endocytosis.

## 2. Results

### 2.1. NT4 Peptide Binds to HS and Is Internalized with HS

**Figure 1:**
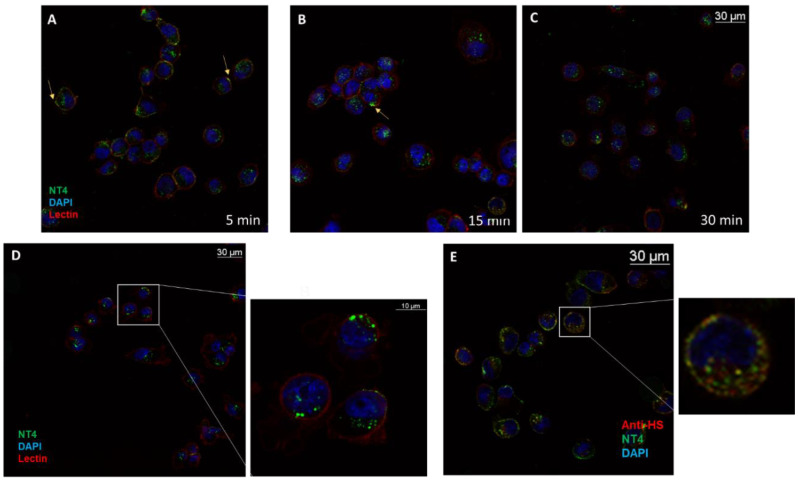
NT4 binds the plasma membrane of MDA-MB-231 and is internalized within 30 min. (**A**) After 5 min, part of the NT4 green signal is on the membrane (yellow arrow); (**B**) after 15 min, the membrane-bound portion of the peptide has decreased; (**C**) after 30 min, most of the peptide is inside the cell, localized in endosomes (**D**); (**E**) anti-HS antibody colocalizes with NT4 on the membrane and in endosomes.

NT4 peptide previously proved to bind heparan sulfate chains in surface plasmon resonance experiments [13] and also to bind to MDA-MB231 cells in flow cytometry experiments [19]. Detailed mapping of NT4 binding on heparan sulfate chains showed a preference for hypersulfated patches of glypicans and syndecans [19]. The peptide bound to the plasma membrane (Figure 1A) [13], most was internalized in cells within 15–30 min (Figure 1B,C) and it was completely endocytosed within 1 h (Figure 1D). The antibody anti-HS clone 10E4 [20], the epitope of which includes an N-sulfated glucosamine residue, was used in a colocalization test with the peptide. The peptide and the antibody colocalized in clusters on the plasma membrane (Figure 1E) and inside the cell, proving that the bound pair is internalized unresolved (Figure 1E). The overlap rate measured with the specific microscope software [21] was 71% (±4.41%; *n* = 9), with a mean Pearson correlation of 0.6561 (±0.03) on three experiments and three randomly taken fields.

### 2.2. NT4 Peptide Internalization Follows More than One Pathway

**Figure 2:**
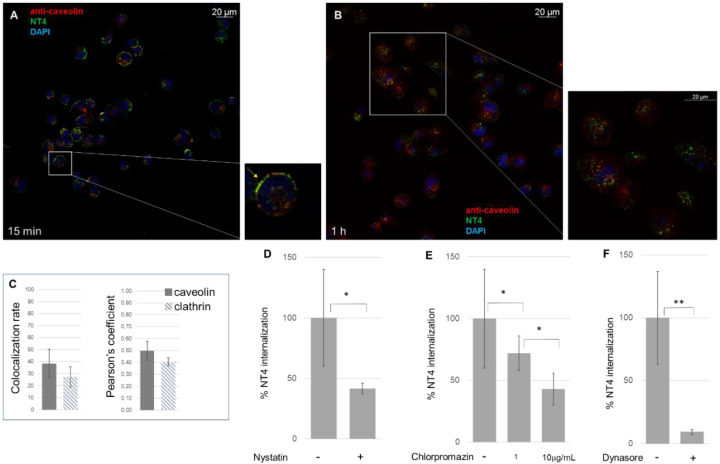
The process of NT4 endocytosis is caveolin-mediated. Colocalization of NT4 with caveolin after (**A**) 15 min, the yellow arrow in the zoomed area shows colocalization, and (**B**) 1 h incubation; (**C**) colocalization rate of NT4 and anti-caveolin (*n* = 12) and anti-clathrin antibodies (*n* = 9), after 30 min incubation, measured as colocalization rate and Pearson coefficient. (**D**) Inhibition of NT4 internalization by nystatin (* *p* = 0.0345, *n* = 9), (**E**) by chlorpromazine 1 μg/mL (* *p* = 0.0279, *n* = 3) and 10 μg/mL (* *p* = 0.0327, *n* = 4) and (**F**) by dynasore (** *p* = 0.0043, *n* = 6).

An anti-caveolin antibody was used in a colocalization experiment with NT4 (Figure 2A). MDA-MB-231 cells were incubated with the peptide at 37 °C for different times: 5, 15 and 30 min and 1 h. The cells were then stained with the caveolin antibody. The colocalization rate in the first 30 min was 38% (±12.03%; *n* = 12) and the Pearson coefficient was 0.4976 (±0.08) (Figure 2A), obtained from three experiments and different randomly selected fields. After 1 h incubation, colocalization with caveolin fell below 30% (Figure 2B).

Partial localization of the NT4 bound to HS in caveolae is consistent with previous studies which found that HSPGs and chondroitin sulfate proteoglycans (CSPGs) colocalized intracellularly with internalized caveolin-1 [22,23].

To confirm the result, nystatin was used as inhibitor of caveolin-mediated endocytosis. Nystatin distorts the structure and functions of cholesterol-rich membrane domains, including caveolae, and is considered a selective inhibitor of the lipid raft and caveolae pathway [24]. MDA-MB-231 cells were incubated for 15 min with nystatin and NT4. Endocytosis was inhibited by 60% (Figure 2D), in line with the fact that the caveolin-mediated pathway is not the only pathway of endocytosis of NT4.

Clathrin-mediated endocytosis was analyzed using an anti-clathrin antibody and chlorpromazine, a cationic amphipathic drug that prevents formation of new clathrin-coated vesicles [25]. The colocalization rate was 28%, with a Pearson coefficient of 0.4051 (±0.1; *n* = 9). The effect of chlorpromazine was evident and dose-dependent, showing reductions of 57% (±13%) and 27% (±13%) with 10 and 1 μg/mL of the inhibitor, respectively. The result is in agreement with previous findings showing that NT4 is also a ligand of LRP6, a co-receptor of low density lipoprotein (LDL) that is internalized via clathrin endosomes [13,26,27].

Dynasore is a specific inhibitor of dynamin [28], a protein that severs vesicles from the plasma membrane during clathrin- and caveolin-dependent endocytic processes [29] and also during macropinocytosis [30]. When treated with dynasore, MDA-MB-231 cells no longer internalized NT4 (Figure 2E). The impairment of internalization was greater than with chlorpromazine and nystatin, consistent with the fact that dynasore hampered both clathrin- and caveolin-mediated endocytosis.

Macropinocytosis is a cell process by which much extracellular material is engulfed. It is driven by actin that induces membrane ruffling and formation of large endosomes, i.e., macropinosomes [31]. We investigated macropinocytosis as a further possible endocytic pathway for HSPG-mediated NT4 internalization. Rhodamine-labelled dextran was used in localization experiments.

**Figure 3:**
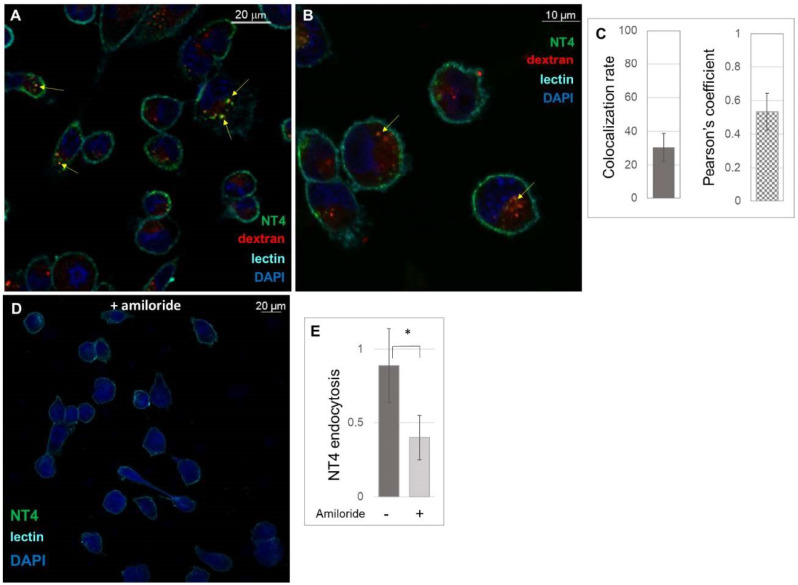
Macropinocytosis of NT4. (**A**) Confocal experiments of colocalization of NT4 (green) and dextran (red); (**B**) higher magnification of a confocal image of colocalization, yellow arrows indicate colocalization of NT4 and dextran; (**C**) measurement of colocalization of NT4 and dextran; (**D**,**E**) pre-treatment of MDA-MB-231 cells with amiloride impairs NT4 endocytosis (* *p* = 0.0461).

NT4 and dextran partially colocalized, 26.44% (±8.31%; *n* = 10) (Figure 3A,B), with a Pearson coefficient of 0.5339 (±0.11) (Figure 3C). Amiloride is an inhibitor of macropinocytosis [32] and can impair NT4 internalization by more than 50% (Figure 3D,E).

### 2.3. Vesicle Trafficking in Triple-Negative Breast Cancer Cells

**Figure 4:**
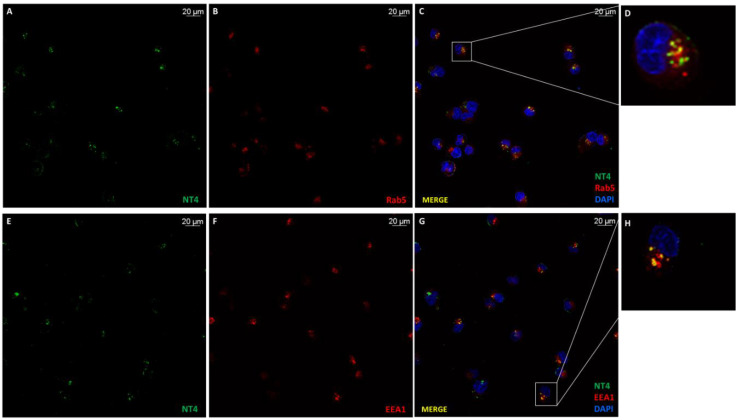
Co-immunostaining of NT4 with Rab5 and EEA1. (**A**) NT4 (green) and (**B**) Rab5 (red) colocalized (**C**,**D**); (**E**) NT4 and EEA1 (**F**) colocalized (**G**,**H**).

NT4 was internalized in a few minutes after membrane-binding, and once in the cytosol, it could be observed in vesicles with HSPG (Figure 1). Hence, trafficking of NT4 is linked to that of HSPGs. Rab proteins are small GTPases that regulate intracellular membrane trafficking with active roles in formation, fusion and movement of vesicles [9,33]. Rab5 controls and regulates the intracellular transport of various receptors [10] and its presence on vesicles defines them as early endosomes. NT4 showed a colocalization rate with Rab5 of 39% (*n* = 11, SD ± 13) and a Pearson coefficient of 0.51 (*n* = 11, SD ± 0.06) in immunofluorescence experiments using a Rab5A antibody (Figure 4A–D).

EEA1 is associated with Rab5 and consistently colocalized with NT4 at a rate of 33% (*n* = 14, SD ± 3) and a Pearson coefficient of 0.4801 (*n* = 14, SD ± 0.0050) (Figure 4E–H).

**Figure 5:**
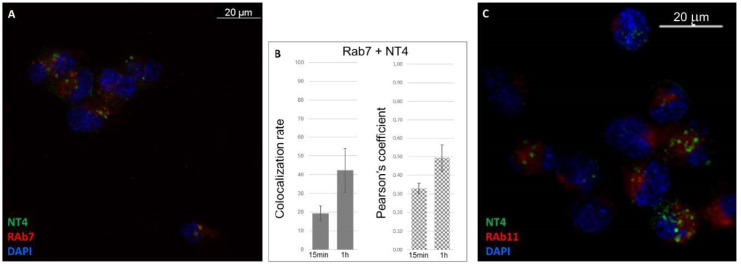
NT4 colocalization with Rab7 and Rab11. (**A**) NT4 and Rab7 after 1 h incubation; (**B**) normalized values of colocalization rate and Pearson coefficient of NT4 and Rab7 after 15 min and 1 h incubation; (**C**) NT4 and Rab11 after 1 h incubation.

Rab7 protein marks late endosomes. The subcellular location of NT4 and Rab7 was observed at two incubation times (15 and 60 min) with the peptide at 37 °C. As expected, colocalization increased in the time interval (Figure 5A,B): after 15 min, it was 19% (*n* = 10, SD ± 4), with a Pearson coefficient of 0.33 (*n* = 10, SD ± 0.03); after 1 h, the rate increased to 42% (*n* = 10, SD ± 11), with a Pearson coefficient of 0.49 (*n* = 10, SD ± 0.07) (Figure 5A,B).

Rab11, a small GTPase associated with endosome-recycling vesicles, gave a colocalization rate of 24% (*n* = 6; SD ± 5.89) and a low Pearson coefficient (0.38; *n* = 6, SD ± 0.039) (Figure 5C), indicating scarce presence in the recycling endosomes.

### 2.4. Effects of NT4 on Cancer Cell Migration

**Figure 6:**
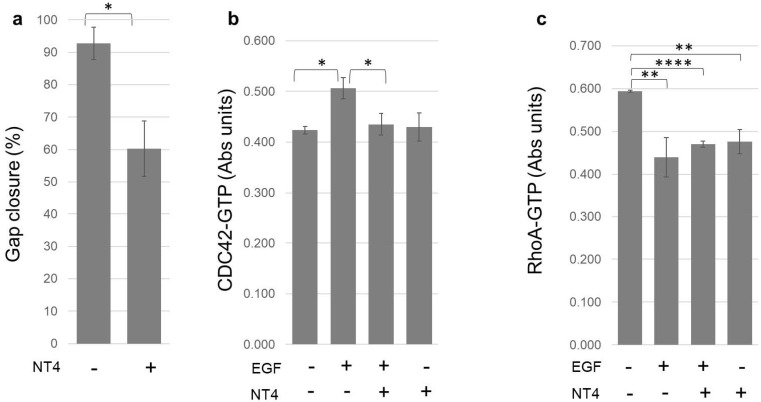
(**a**) MDA-MB-231 migration measured as gap closure in 8 h in a scratch assay (* *p* = 0.0428, *n* = 2); (**b**) effect of NT4 on CDC42 activation (*n* = 2; * *p* = 0.0196 and * *p* = 0.0132); (**c**) effect of NT4 on RhoA activation (*n* = 3; ** *p* = 0.0043, **** *p* < 0.0001, and ** *p* = 0.0020).

The polycationic peptide NT4 has already been shown to interfere with cancer cell migration [34], impairing 2D and 3D migration of pancreas cancer cells and HUVEC endothelial cells and hampering invasive migration of MDA-MB-231 cells through collagen [18]. In a standard 2D migration experiment, NT4 slowed MDA-MB-231 migration in a wound-scratch experiment (Figure 6a). Cell migration is closely linked to cytoskeletal rearrangements and vesicle trafficking, cell events regulated by Rho GTPases [35].

We hypothesized that NT4 could interfere with Rho-GTPases, particularly CDC42, Rac-1 and RhoA, which have well-established roles in regulating actin polymerization and, thus, migration and endocytosis. Activation of CDC42, Rac1 and RhoA was stimulated by EGF in starved MDA-MB-231 cells.

G-LISA experiments [7] showed that CDC42-GTP is increased by EGF and that NT4 mitigates activation of CDC42 induced by the growth factor [36]; on the other hand, NT4 without EGF has no effect on CDC42 activation (Figure 6b).

Rac1-GTP was transiently activated by EGF in G-LISA experiments, as already described by other authors [37]. The activation was not significantly changed by NT4. Besides, basal levels of Rac-1-GTP, which are very low in MDA-MB-231 [38], were not affected by NT4 in a pulldown experiment (Supplementary Materials).

RhoA-GTP was decreased by EGF, as already observed by other authors [38,39], and NT4 did not significantly interfere with this effect. RhoA was reduced by EGF, NT4 and EGF with NT4 of 26%, 20% and 21%, respectively; the three groups showed no statistical differences—in other words, NT4 and EGF do not compete or synergize in RhoA activation. Besides, NT4 itself reduced the basal level of activated RhoA, even without EGF, thus with no interference with the growth factor function.

## 3. Discussion

NT4 binds HSPGs on the plasma membrane and is internalized with HSPG in endosomes. Internalization occurred by different pathways; caveolin- and clathrin-dependent endocytosis and macropinocytosis were all employed by the peptide, in line with what we already know about HSPGs [2,3,4,5,6,7,8]. NT4 then localized in early and late endosomes in a time-dependent manner.

The polycationic peptide had previously been shown to impair migration of MDA-MB-231 and other cancer cell lines in 2D and 3D models. Our hypothesis was that HSPG endocytosis could affect actin organization, influencing two important phenomena of cancer development: movement and nourishment. Using NT4 to mimic heparin-binding ligands, we studied the effect of Rho-GTPases, specifically CDC42, Rac1 and RhoA.

**Figure 7:**
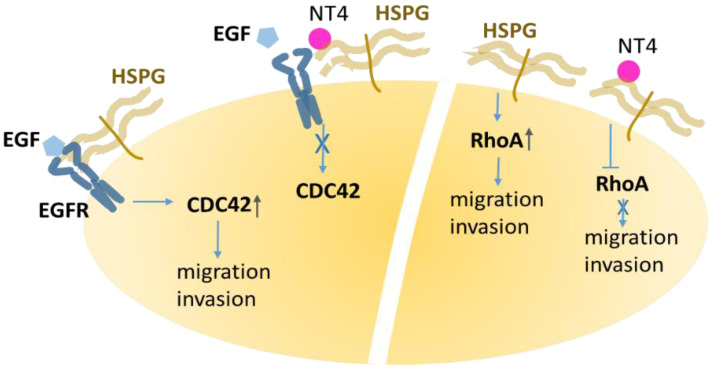
EGF binding to the receptor, assisted by heparan sulfate proteoglycans (HSPGs), triggers CDC42 activation. NT4 binding to HSPG disrupts the EGF-EGFR-HSPG triad and reduces CDC42 activation. Besides, NT4 dampens RhoA activation, without interference with EGF-EGFR systems, confirming the ability of HSPGs to directly modulate this GTPase.

CDC42 is a small GTPase, the multiple roles of which have been abundantly described. Its downstream effectors are various kinases, such as PAKs, MLKs, and MRCKs [40], which initiate processes such as cell polarity [41], cytoskeletal remodeling, migration, adhesion, membrane trafficking, proliferation and transcription [40]. Activated by EGF, CDC42 promotes actin branching in invadopodia, using its effector N-WASP and, in turn, Arp2/3 [37]. NT4 shared localization with HSPGs and had a negative effect on CDC42-activation triggered by EGF. The effect is presumably due to competitive inhibition of EGF on HS by NT4, which impairs the co-receptor activity of the proteoglycan and reduces EGFR activation (Figure 7). The reduction of the invasive and migratory phenotype of MDA-MB-231, induced by NT4, can be ascribed to this effect.

RhoA is a member of the Rho GTPase family and, as with Rac1 and CDC42, is often described as having a role downstream of EGF activation and also as promoting carcinogenesis and metastasis [37]. In fact, RhoA activation is damped by EGF in MDA-MB-231 [42], as also confirmed by our findings. Indeed, EGF reduced RhoA-GTP and the HS-binding peptide did not interfere with this receptor-mediated signaling. On the other hand, the peptide itself determined a small but solid reduction in active RhoA in breast cancer cells (Figure 6 and Figure 7). Recently, a role of syndecans, not involving growth factor activation but rather the cytoplasmic domains of transmembrane syndecans that can act as signaling receptors, was observed in RhoA activation [43]. Our result, showing that NT4 dampens RhoA activation, a key step for cell movement, correlates well with the peptide anti-migratory activity. The observation that the HSPG-binding peptide NT4 down-regulates active RhoA without antagonizing EGF, is a reasonable confirmation that RhoA is directly activated through HSPG [43].

In conclusion, our data suggest that HSPGs are, as other authors observed, internalized following different pathways and are localized in specific endosomes. Further studies are necessary to understand possible roles of HSPGs in vesicle transport, not only by virtue of their ability to capture exosomes [44], which is well described, but also through regulating endosome traffic, which is vital for cell movement and nourishment. Besides, the results support the observation of few other studies, showing that HSPGs are not simple co-receptors that promote or prevent ligand binding to their receptors, as they were considered for many years, but have a direct role in signaling.

## 4. Materials and Methods

### 4.1. Peptide Synthesis

Peptides were synthesized by standard Fmoc chemistry on an automated multiple synthesizer (MultiSynTech, Witten, Germany). NT4 was synthesized on Fmoc4-Lys2-Lys-beta-Ala-Wang resin, using protected L-amino acids (Iris Biotech Marktredwitz, Germany), DIPEA (N,N-diisopropylethylamine) (Merck) and HBTU (O-benzotriazole-N,N,N’,N’-tetramethyl-uronium-hexafluoro-phosphate) (Iris Biotech). Pyro-Glu-O-pentachlorophenylester (Bachem, Bubendorf, Switzerland) was used for the last coupling step. NT4-biotin was synthesized on Tentagel resin using Fmoc-Lys(biotin)-OH and Fmoc-Lys(Tmr)-OH (Invitrogen, Carlsbad, CA, USA), respectively, as the first and Fmoc-PEG12-OH as the second coupling step; Fmoc-Lys(Fmoc)-OH was then used to build the tetrameric core. At the end of the coupling sequence, peptides were cleaved from the resin, deprotected and lyophilized.

HPLC purification was performed on a C18 Jupiter column (Phenomenex, Torrance, CA, USA). Water with 0.1% TFA (A) and acetonitrile (B) were used as eluents. Linear gradients over 30 min were run at flow rates of 0.8 and 4 mL/min for analytical and preparatory procedures, respectively.

All compounds were also characterized on a Bruker Ultraflex MALDI TOF/TOF mass spectrometer.

NT4 (pyELYENKPRRPYIL)_4_K_2_K-beta-Ala. MS: m/z calculated for C333H519N91O81 [M+H]^+^ 7094.24; detected 7095.15. HPLC RT (from 80%A to 20%A) was 26.63 min.

NT4-biotin (pyELYENKPRRPYIL)_4_K_2_K-PEG_12_-K(biotin). MS: m/z calculated for C373H594N96O95S [M+ H]^+^ 7976.35; detected 7978.72. HPLC RT (from 80%A to 20%A) was 26.99 min.

### 4.2. Cell Cultures

MDA-MB-231 human breast adenocarcinoma cells were grown in their recommended medium: Dulbecco’s Modified Eagle Medium (DMEM) supplemented with 10% fetal bovine serum, 200 μg/mL glutamine, 100 μg/mL streptomycin and 60 μg/mL penicillin at 37 °C, 5% CO_2_. The cell line was purchased from ATCC (Manassas, VA, USA).

### 4.3. Immunofluorescence

MDA-MB-231 cells were plated at a density of 5 × 10^4^ per well in 24-well plates with glass cover slides and maintained overnight at 37 °C, 5% CO_2_.

#### 4.3.1. Detection of NT4 Binding to HS and Internalization with HS

Cells were incubated with 2 μM NT4-biotin and 0.5 μg/mL Streptavidin-Atto 488 in PBS-1% BSA at room temperature for 15 min, then washed and grown in medium for 5, 15 and 30 min at 37 °C to allow peptide internalization. They were then fixed with PBS-4% paraformaldehyde (PFA) and saturated with PBS-5% BSA 0.3%-Triton X-100. HS was stained using 1 µg/mL anti-heparan sulfate (10E4 epitope) (Amsbio, Abingdon, UK) in PBS-1% BSA for 1 h, followed by incubation with 1 μg/mL anti-mouse IgG Rhodamine Red-X (Thermo Fisher Scientific, Waltham, MA, USA). Nuclei were stained with DAPI (0.5 μg/mL in PBS-1% BSA). Each step was followed by three washes in PBS. Peptide binding was analyzed by confocal laser microscope (Leica TCS SP5) with 380 λ ex and 450–470 λ em, 501 λ ex and 523 λ em and 633 λ ex and 660–680 λ em for DAPI, Atto 488 and Atto 647, respectively. The experiment was repeated three times and three different fields were acquired for each sample.

#### 4.3.2. Detection of NT4 Internalization in Presence of Pharmacological Agents

Cells were pretreated for 15 min with 1–10 μg/mL Chlorpromazine (Merck, Darmstadt, Germany), 25 μg/mL Nystatin (Merck), 80 μM Dynasore (Merck) and 50 µM Amiloride (Merck) at 37 °C and then incubated with 2 μM NT4-biotin and 0.5 μg/mL Streptavidin-Atto 488 in PBS-1% BSA at room temperature for 15 min. To study macropinocytosis in the presence of Amiloride, 5 mg/mL Rhodamine B isothiocyanate–Dextran in PBS-1% BSA (Merck) was incubated at room temperature for 15 min. Cells were then washed and grown in medium, with and without pharmacological agents, for 15 and 30 min at 37 °C to allow peptide internalization. After internalization, they were fixed with PBS-4% PFA and plasma membranes were stained with 2.5 μg/mL wheat germ agglutinin, Alexa Fluor 647 (Thermo Fisher Scientific) conjugate in PBS-1% BSA, incubated for 10 min at room temperature. Nuclei were stained with DAPI (0.5 μg/mL in PBS-1% BSA). Each step was followed by three washes in PBS.

Peptide binding was analyzed by confocal laser microscope (Leica TCS SP5) with 501 λ ex and 523 λ em for Atto 488, 633 λ ex and 660–680 λ em for Atto 647 and 364 λ ex and 458 λ em for DAPI. Images were single Z-planes for all channels used. All images were processed and quantified using the Leica Application Suite X program (LAS X) of the confocal laser microscope (Leica TCS SP5). The experiment was repeated at least three times and at least three different fields were acquired for each sample.

#### 4.3.3. Detection of NT4 Internalization in Presence of Endosomal Markers

Cells were incubated with 2 μM NT4-biotin and 0.5 μg/mL Streptavidin-Atto 488 in PBS-1% BSA at room temperature for 15 min. The cells were then washed and grown in medium for 5, 15 and 30 min and 1 h at 37 °C to allow peptide internalization. After internalization, they were fixed with PBS-4% PFA, permeabilized with PBS-0.3% Triton X-100 for 10 min (only for Rab5), saturated with PBS-5% BSA 0.3%- Triton X-100 for 1 h and then incubated at 4 °C overnight with endosomal antibodies (Cell Signaling) diluted in PBS-1% BSA 0.3% Triton X-100: caveolin, diluted 1:400; Rab5, diluted 1:400; Rab7, diluted 1:100; clathrin, diluted 1:50; Rab11, diluted 1:100; EEA1, diluted 1:100.

Cells were finally incubated at room temperature for 1 h with secondary antibodies: for Rab5, anti-mouse IgG Rhodamine Red-X (Thermo Fisher Scientific), diluted 1:1000 PBS-1% BSA 0.3% Triton X-100; for Rab7, Rab11, EEA1, caveolin and clathrin, anti-rabbit IgG Alexa Fluor 546 (Life Technologies, Carlsbad, CA, USA), diluted 1:1000 in PBS-1% BSA 0.3% Triton X-100. Nuclei were stained with DAPI (Sigma-Aldrich, St. Louis, MO, USA) (0.5 μg/mL in TBS-1% BSA) 380 λ ex and 450–470 λ em.

Samples were analyzed by confocal laser microscope (Leica TCS SP5) with 364 λ ex and 458 λ em for DAPI, 501 λ ex and 523 λ em for Atto 488, 560 λ ex and 580 λ em for Rhodamine Red-X and 556 λ ex and 576 λ em for Alexa Fluor 546. All images were processed and quantified using the Leica Application Suite X program (Las X) of the confocal laser microscope (Leica TCS SP5). The experiment was repeated three times and three different fields were acquired for each sample.

### 4.4. D Migration

Cell migration was measured using an in vitro wound-healing assay. MDA-MB231 cells (5.25 × 10^4^) were seeded on each side of a culture insert for live cell analysis (Ibidi, Munich, Germany). Inserts were placed in the wells of a 24-well plate and the cells were incubated at 37 °C and 5% CO_2_ to confluence. The inserts were removed with sterile tweezers to create a cell-free area of approximately 500 μm. The cells were treated with 10 μM NT4 peptide in complete medium and allowed to migrate in an appropriate incubator of a DMi8 (Leica Microsystems, Wetzlar, Germany) microscope. The same instrument was used to take pictures at time zero and every 5 min for 11 h.

The percentage of void area with respect to time 0 was determined using Tscratch. The time-lapse image stacks were also analyzed using ImageJ and the plug-in Chemotaxis and Migration Tool.

### 4.5. G-LISA

Cells were seeded in six-well plates (5 × 10^5^ cells per well) and maintained for 12 h in a CO_2_ incubator. To measure Rac-1, RhoA and CDC42 GTPases, they were treated with 10 µM NT4 in DMEM-0.1% BSA, serum-free, for 12 h at 37 °C and then incubated with 0.1 μg/mL hEGF (Cell Signaling) for 2 min for Rac-1 and CDC42 GTPases and for 5 min for RhoA GTPase. Cells were washed with cold PBS, lysed and processed for colorimetric G-LISA activation assays (Cytoskeleton, Denver, CO, USA), according to the manufacturer’s instructions. The activated forms of the G proteins were detected by incubation with specific primary antibody, followed by a secondary antibody conjugated with HRP and a detection reagent. The signal was read by measuring absorbance at 490 nm using a microplate reader. The experiment was repeated three times.

### 4.6. Pulldown Assay

Rac1 activity was measured by pulldown assay using the active Rac1 detection kit (Cell Signaling), according to the manufacturer’s instructions. Cells were seeded in six-well plates (5 × 10^5^ cells per well) and maintained for 24 h in a CO_2_ incubator. Cells were washed with cold PBS and lysed. Protein lysates were centrifuged and the supernatant was collected in new tubes containing beads pre-coupled with GST–PAK1-PBD and incubated under rotation at 4 °C for 60 min. The beads were washed and the proteins bound to them were separated by SDS-PAGE. The amounts of active Rac1 were determined by immunoblot analysis. Signals were detected using a LAS4010 imaging system (GE Healthcare, Chicago, IL, USA).

### 4.7. Statistical Data

All experiments were repeated at least three times and the data were presented as mean ±SD, accompanied with the number of samples (*n*). The significance of differences between groups was analyzed by one-tailed Student’s *t*-test using GraphPad Prism 5.03 software; *p* values are reported in figure legends.

## Supplementary Materials

Supplementary materials can be found at https://www.mdpi.com/1422-0067/21/21/8282/s1.
