# Supplementary material for: Endocytosis and Trafficking of Heparan Sulfate Proteoglycans in Triple-Negative Breast Cancer Cells Unraveled with a Polycationic Peptide
Source: Int J Mol Sci. 2020 Nov 5;21(21):8282. doi: 10.3390/ijms21218282 (PMC7663799; doi:10.3390/ijms21218282)
Supplement: Supplementary file 1 [file ijms-21-08282-s001.pdf]

Supplementary Materials

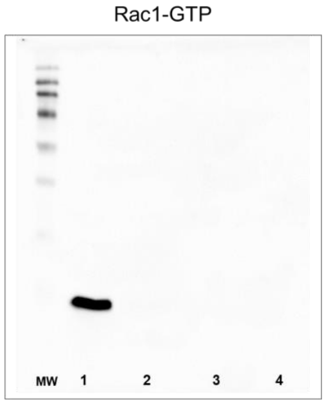

Figure 1SM. Pull-down assay of Rac1-GTP in MDA-MB-231. Lane 1: positive control; lane 2: negative control, lane 3: untreated cells (control); lane 4: cells treated with NT4.
